# Comparative transcriptome analysis identifies crucial candidate genes and pathways in the hypothalamic-pituitary-gonadal axis during external genitalia development of male geese

**DOI:** 10.1186/s12864-022-08374-2

**Published:** 2022-02-15

**Authors:** Bincheng Tang, Shenqiang Hu, Qingyuan Ouyang, Tianhao Wu, Yao Lu, Jiwei Hu, Bo Hu, Liang Li, Jiwen Wang

**Affiliations:** Farm Animal Genetic Resources Exploration and Innovation Key Laboratory of Sichuan Province, Chengdu Campus, Sichuan Agricultural University, Wenjiang District, Chengdu, 611130 Sichuan China

**Keywords:** Goose, HPG axis, External genitalia development, Transcriptome sequencing

## Abstract

**Background:**

All birds reproduce via internal fertilization, but only ~3% of male birds possess the external genitalia that allows for intromission. Waterfowl (e.g., duck and goose) are representatives of them, and the external genitalia development of male geese is directly related to mating ability. Notably, some male geese show abnormal external genitalia development during ontogenesis. However, until now little is known about the molecular mechanisms of the external genitalia development in goose. In the present study, comparative transcriptomic analyses were performed on the hypothalamus, pituitary gland, testis, and external genitalia isolated from the 245-day-old male Tianfu meat geese showing normal (NEGG, *n* = 3) and abnormal (AEGG, *n* = 3) external genitals in order to provide a better understanding of the mechanisms controlling the development of the external genitalia in aquatic bird species.

**Results:**

There were 107, 284, 2192, and 1005 differentially expressed genes (DEGs) identified in the hypothalamus, pituitary gland, testis and external genitalia between NEGG and AEGG. Functional enrichment analysis indicated that the DEGs identified in the hypothalamus were mainly enriched in the ECM-receptor interaction pathway. The ECM-receptor interaction, focal adhesion, and neuroactive ligand-receptor interaction pathways were significantly enriched by the DEGs in the pituitary gland. In the testis, the DEGs were enriched in the neuroactive ligand-receptor interaction, cell cycle, oocyte meiosis, and purine metabolism. In the external genitalia, the DEGs were enriched in the metabolic, neuroactive ligand-receptor interaction, and WNT signaling pathways. Furthermore, through integrated analysis of protein-protein interaction (PPI) network and co-expression network, fifteen genes involved in the neuroactive ligand-receptor interaction and WNT signaling pathways were identified, including *KNG1*, *LPAR2*, *LPAR3*, *NPY*, *PLCB1*, *AVPR1B*, *GHSR*, *GRM3*, *HTR5A*, *FSHB*, *FSHR*, *WNT11*, *WNT5A*, *WIF1*, and *WNT7B*, which could play crucial roles in the development of goose external genitalia.

**Conclusions:**

This study is the first systematically comparing the hypothalamus, pituitary gland, testis, and external genitalia transcriptomes of male geese exhibiting normal and abnormal external genitals. Both bioinformatic analysis and validation experiments indicated that the neuroactive ligand-receptor interaction pathway could regulate the WNT signaling pathway through *PLCB1* to control male goose external genitalia development.

**Supplementary Information:**

The online version contains supplementary material available at 10.1186/s12864-022-08374-2.

## Background

All birds reproduce via internal fertilization, but only ~3% of male birds retain the external genitalia capable of intromission [1]. In poultry, most of terrestrial fowl (e.g., chicken) lack the intromittent external genitalia [2], while waterfowl (e.g., duck and goose) retain the well-developed external genitalia [3]. During the internal fertilization process, the external genitalia of male poultry inserts into the female’s cloaca to deliver sperm [4]. In waterfowl, the external genitalia size and structural characteristics (e.g., smoothness and percentage of coverage by spines and grooves) are closely related to the frequency of copulations [5]. It has also been shown that the extent of the external genitalia development could affect the insemination capacity of male geese [6].

Over the past few years, studies in both mammals and birds have identified some candidate genes related to external genitalia development through investigations of their mRNA expression levels. For instance, the external genitalia was absent in mice containing a targeted deletion of sonic hedgehog (*Shh*) [7]. In marsupials, expression of insulin growth factor 1 (*IGF1*) was upregulated during the external genitalia growth and elongation [8]. Herrera et al. (2013) found that the evolutionary reduction of the external genitalia in birds occurred via *de novo* activation of cell death elicited by bone morphogenetic protein 4 (*BMP4*) in the genital tubercle [1]. In male geese, the 17α-hydroxylase/17, 20-lyase (*CYP17*) gene plays a key role during the external genitalia development [9]. Nevertheless, the regulatory mechanism controlling goose external genitalia development still remains unclear.

The hypothalamic-pituitary-gonadal (HPG) axis is a coordinated neuroendocrine system that regulates the development of poultry reproductive system [10, 11]. Recently, the emerging omic studies on the HPG axis have revealed candidate genes and pathways that may have important roles in regulating reproductive traits in poultry. For example, transcriptome sequencing of the hypothalamus and pituitary gland from chickens with different egg numbers revealed an important role of the steroid hormone biosynthesis pathway in the regulation of egg production performance [12]. Meanwhile, the mTOR signaling and PI3K-Akt signaling pathways have also been identified by transcriptome sequencing of chickens showing different egg production performance [13]. Additionally, the DEGs identified in the hypothalamus and pituitary gland may be involved in broodiness in female geese [11]. However, such studies have been scarcely carried out in male geese regarding the mechanisms controlling the external genitalia development.

Next-generation sequencing (NGS) technology has provided a powerful, highly reproducible, and cost-efficient tool for transcriptomic studies [14, 15]. To date, RNA-seq has been used for analysis of reproductive traits in poultry, including laying performance [16] and gonadal development [17]. Nevertheless, changes in the mRNA expression profiling in the HPG axis during male goose external genital development have not been reported. Therefore, this study aimed to analyze and compare the transcriptome profiles of the hypothalamus, pituitary gland, testis, and external genitalia between NEGG and AEGG geese using RNA-seq. These data were expected to help elucidate the molecular mechanisms regulating male goose external genitalia development.

## Results

### Morphological differences in the external genitalia between NEGG and AEGG

**Fig. 1:**
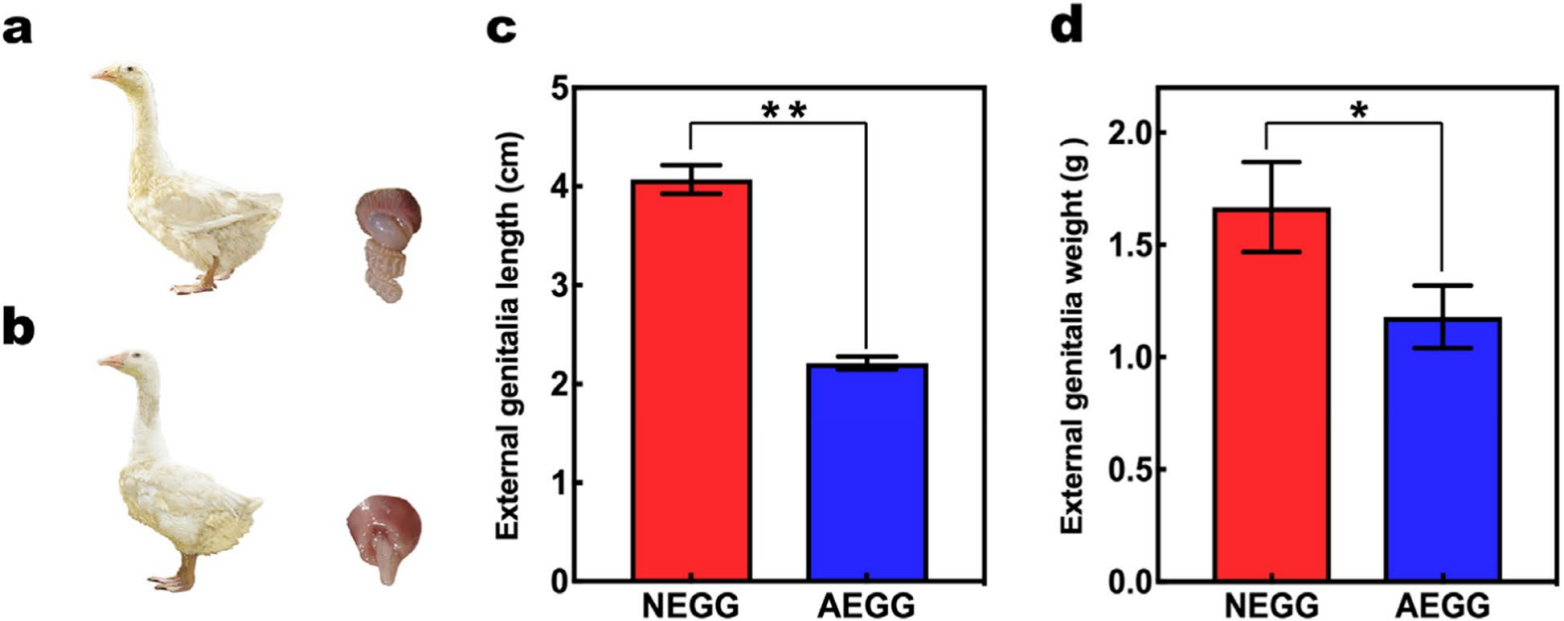
Morphological differences in the external genitalia between NEGG and AEGG geese. **a** Normal external genitalia group geese. **b** Abnormal external genitalia group geese. **c** The length of external genitalia. **d** The weight of external genitalia

As shown in Fig. 1, male geese in the NEGG group showed an elongated and coiled external genitalia with dermal spines (Fig. 1a), whereas those in the AEGG group had a smooth external genitalia (Fig. 1b). In addition, compared with the AEGG group, the length of the external genitalia was significantly longer in the NEGG group (*p*< 0.01, Fig. 1c), and its weight was significantly heavier in the NEGG group (*p*< 0.05, Fig. 1d).

### Overview of transcriptome sequencing

A total of 635,434,075 raw reads were obtained from 24 samples through RNA-seq. Averagely, each sample yielded about 25,138,908 clean reads after strict filtering. The Q20 (percentage of reads with a Phred quality value > 20) and Q30 (percentage of reads with a Phred quality value > 30) of these clean reads ranged from 97.18~97.63% and 92.83~93.63%, respectively. The mapping rate of the clean reads from 24 samples ranged from 74.32~83.64% (Additional file 1: Table S1).

### Identification of the DEGS in the hypothalamus, pituitary gland, testis, and external genitalia between NEGG and AEGG

**Fig. 2:**
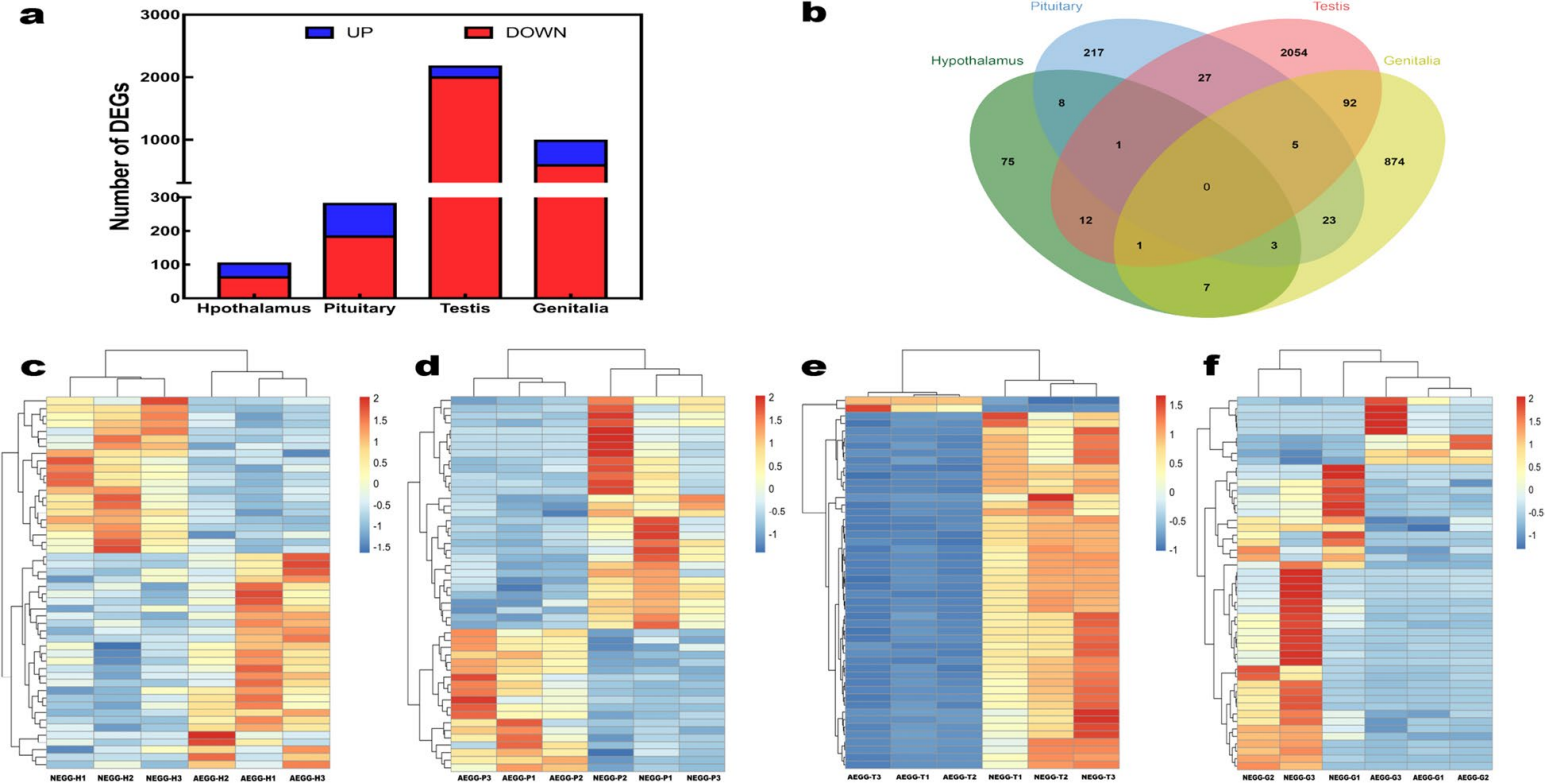
Transcriptomic changes for each tissue and each group. **a** The number of DEGs in different tissues. **b** Venn diagram of the number of DEGs between NEGG and AEGG. **c-f** Pearson’s correlation matrix for the mRNA profiles

We identified 107 DEGs in the hypothalamus, including 41 up-regulated and 66 down-regulated genes. In the pituitary gland, 284 DEGs were identified, and of them 97 were up-regulated and 187 were down-regulated. In the testis, 2192 DEGs were identified, and of them 176 were up-regulated and 2016 were down-regulated. In the external genitalia, 1005 DEGs were identified, and of them 394 were up-regulated and 611 were down-regulated (Fig. 2a; Additional file 2: Fig. S1; Additional file 3: Table S2). A Venn diagram showed that one DEG was commonly identified in the hypothalamus, pituitary gland, and testis between two groups, eight DEGs was commonly identified in the hypothalamus and pituitary gland between two groups, and 92 DEGs were commonly identified in the testis and external genitalia between two groups (Fig. 2b). The hierarchical clustering map (Fig. 2c-f) also recapitulated the different gene expression patterns in the hypothalamus, pituitary gland, testis, and external genitalia between NEGG and AEGG.

### Functional enrichment analysis of the DEGs identified between NEGG and AEGG

**Fig. 3:**
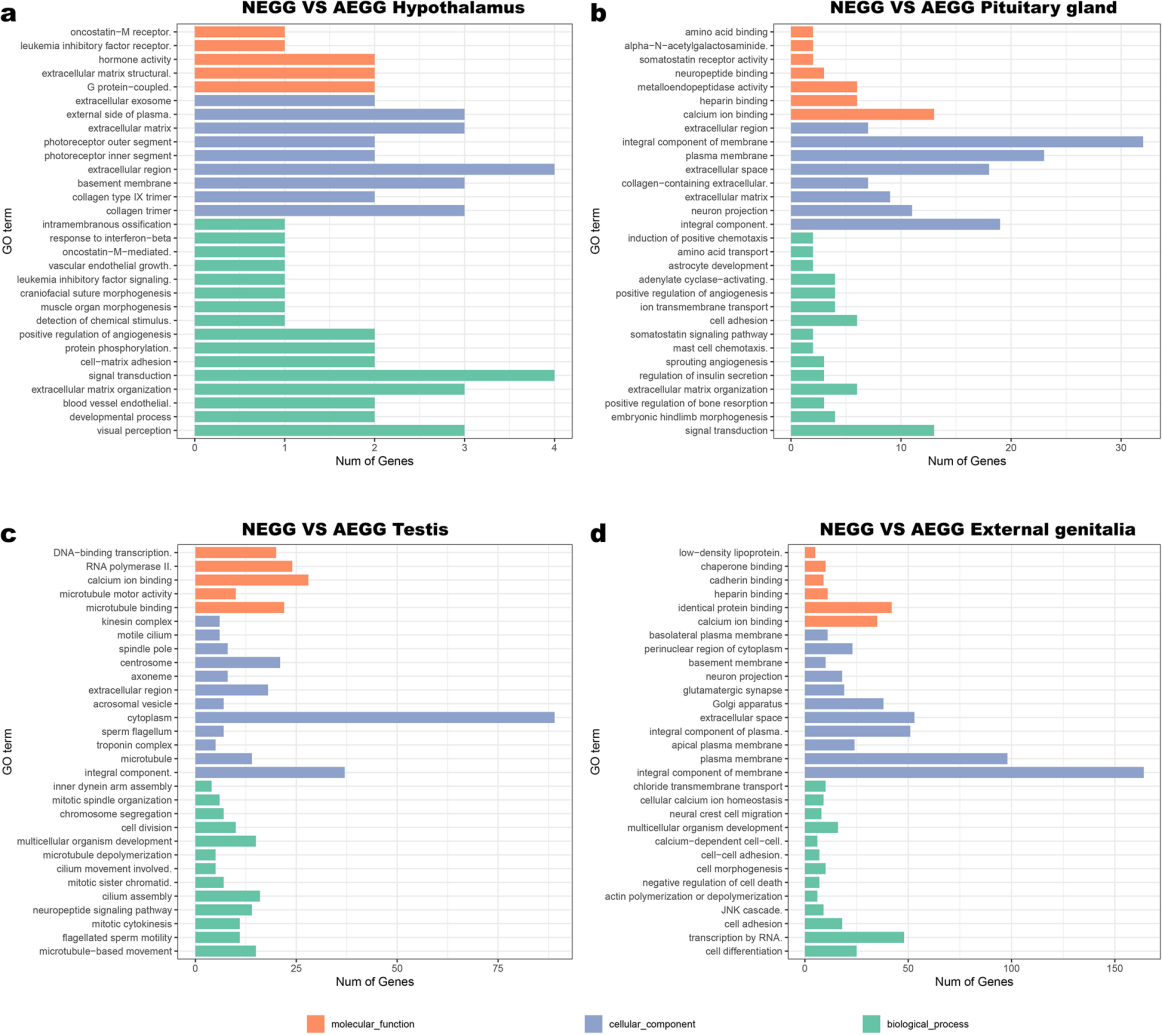
GO terms enriched by DEGs. **a** NEGG-vs-AEGG hypothalamus; **b** NEGG-vs-AEGG pituitary gland; **c** NEGG-vs-AEGG testis; **d** NEGG-vs-AEGG external genitalia. The results were summarized in three main categories: biological process (BP), cellular component (CC), and molecular function (MF). The x-axis indicates the number of genes, and the y-axis indicates the second-level GO term

The DEGs identified in the hypothalamus, pituitary gland, testis, and external genitalia between NEGG and AEGG were further annotated with GO database into three categories, including the biological process (BP), cellular component (CC), and molecular function (MF) (Additional file 4: Table S3). In the hypothalamus, most of DEGs were enriched in collagen trimer (CC), basement membrane (CC), and developmental process (BP) (Corrected *p* <0.05) (Fig. 3a). In the pituitary gland, most of DEGs were enriched in integral component of plasma membrane (CC), neuron projection (CC), signal transduction (BP), extracellular matrix (CC), and calcium ion binding (MF) (Corrected *p* <0.05) (Fig. 3b). In the testis, most of DEGs were enriched in microtubule-based movement (BP), microtubule binding (MF), flagellated sperm motility (BP), mitotic cytokinesis (BP), neuropeptide signaling pathway (BP), microtubule motor activity (MF), and integral component of plasma membrane (CC) (Corrected *p* <0.05) (Fig. 3c). In the external genitalia, most of DEGs were enriched in integral component of membrane (CC), extracellular space (CC), plasma membrane (CC), cell surface (CC), calcium ion binding (MF), integral component of plasma membrane (CC), cell differentiation (BP), cell surface receptor signaling pathway (BP), and canonical WNT signaling pathway (Corrected *p* <0.01) (Fig. 3d).

**Fig. 4:**
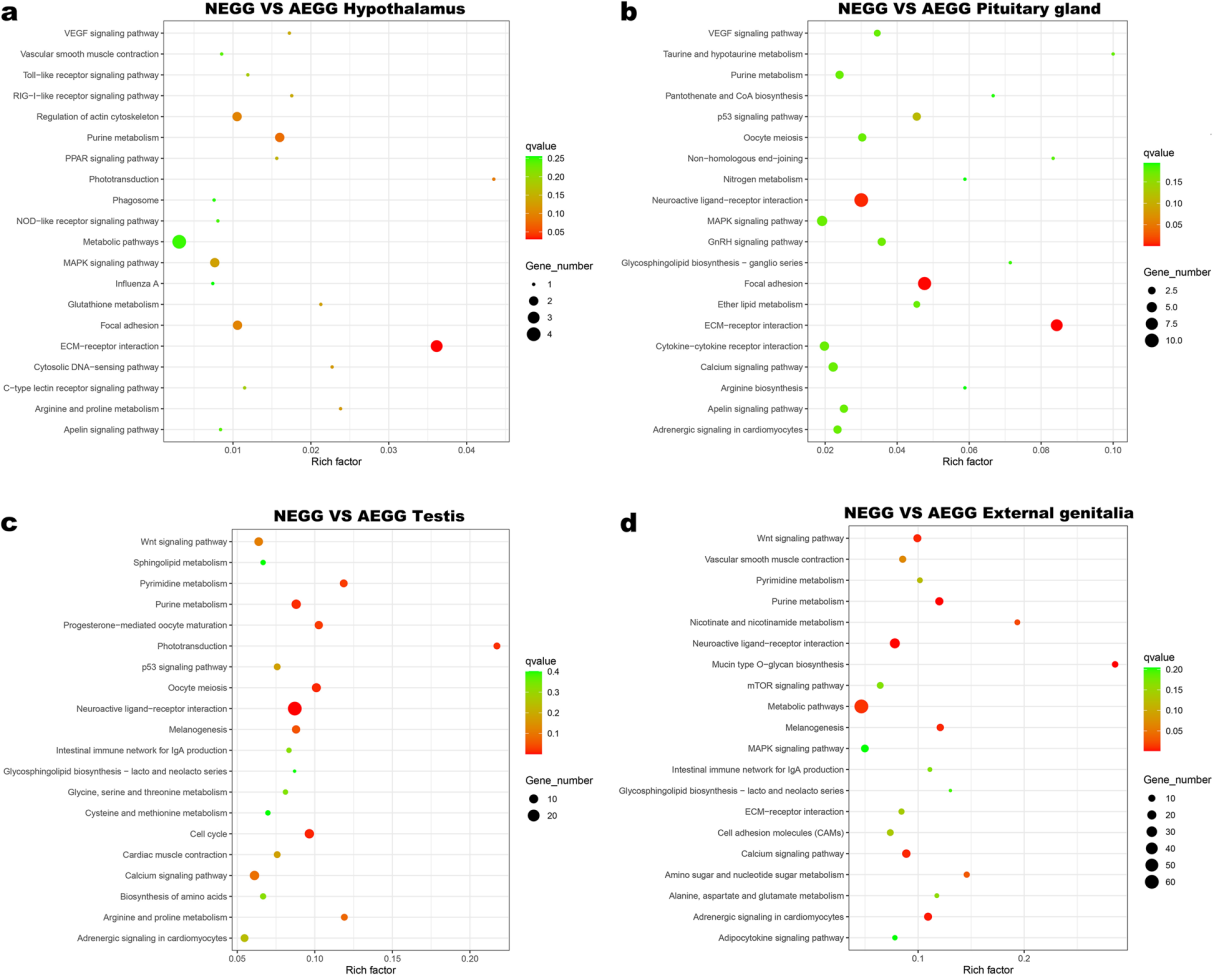
Top 20 significantly enriched KEGG pathways. **a** NEGG-vs-AEGG hypothalamus; **b** NEGG-vs-AEGG pituitary gland; **c** NEGG-vs-AEGG testis; **d** NEGG-vs-AEGG external genitalia. The Rich factor is the ratio of the number of DEGs in the pathway and the total number of genes in the pathway. The higher the Rich factor, the higher is the degree of enrichment. The *q*-value is the *P*-value after multiple hypothesis test correction, in the range from 0 to 1; the closer the *q*-value is to zero, and the more significant is the enrichment

Subsequently, KEGG enrichment analysis showed that a total of 23, 56, 104, and 99 KEGG pathways were enriched in the hypothalamus, pituitary gland, testis, and external genitalia, respectively (Additional file 5: Table S4). The top 20 significantly enriched KEGG pathways were listed in Fig. 4. In the hypothalamus, the most enriched KEGG pathways were ECM-receptor interaction, purine metabolism, and MAPK signaling pathway (Fig. 4a). In the pituitary gland, the five most enriched pathways were ECM-receptor interaction, focal adhesion, neuroactive ligand-receptor interaction, p53 signaling, and GnRH signaling pathways (Fig. 4b). The top 7 significantly enriched KEGG pathways were neuroactive ligand-receptor interaction, cell cycle, oocyte meiosis, purine metabolism, progesterone-mediated oocyte maturation, calcium signaling, and WNT signaling pathways in the testis (Fig. 4c). In the external genitalia, the seven most enriched pathways were metabolic, neuroactive ligand-receptor interaction, calcium signaling, WNT signaling, adrenergic signaling in cardiomyocytes, ECM-receptor interaction, and purine metabolism pathways (Fig. 4d). Notably, the neuroactive ligand-receptor interaction pathway was commonly enriched by the DEGs in the pituitary gland, testis, and external genitalia between NEGG and AEGG.

### Construction of co-expression networks of the DEGs identified between NEGG and AEGG

**Fig. 5:**
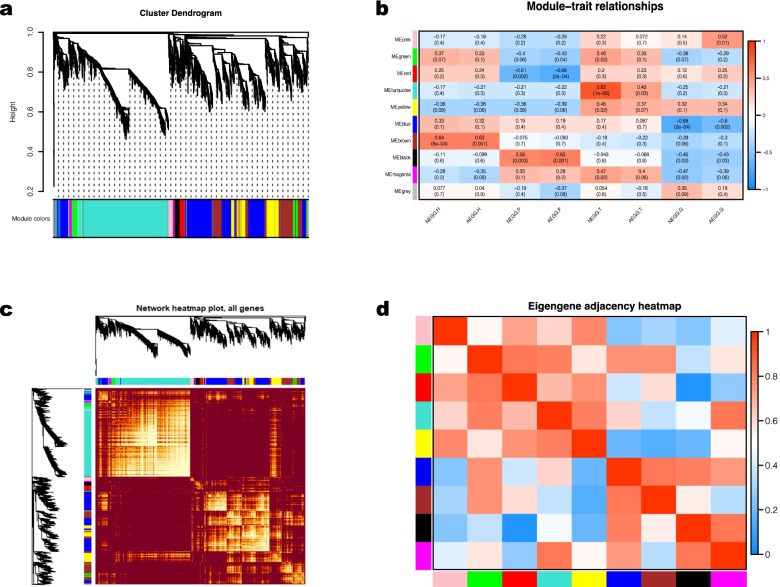
Results of weighted gene co-expression network analysis (WGCNA). **a** Hierarchical clustering tree. Different colors on the abscissa represent different clustering modules. **b** Correlation between modules and traits. The abscissa represents different trait groups, and the ordinate represents different modules. **c** Visualized network heat map. **d** Correlation diagrams between modules. The redder the color of the area where different modules intersect is, the stronger the correlation

The weighted gene co-expression network analysis (WGCNA) was performed to construct the co-expression networks of DEGs in the hypothalamus, pituitary gland, testis, and external genitalia between NEGG and AEGG. The genes with similar expression patterns were classified into different modules (Fig. 5a) (Additional file 6: Table S5). Then, correlation analysis between the modules and phenotypes showed that the blue and brown module had a strong correlation with external genitalia development (Fig. 5b). Meanwhile, correlation analysis between these modules was also performed, and the results indicated that the gene expression pattern in blue module was similar to that in brown module (Fig. 5c, d). Thus, DEGs in the blue and brown modules were selected for subsequent analysis.

### Network analysis and qRT-PCR validation of the DEGs involved in regulating the external genitalia development

**Fig. 6:**
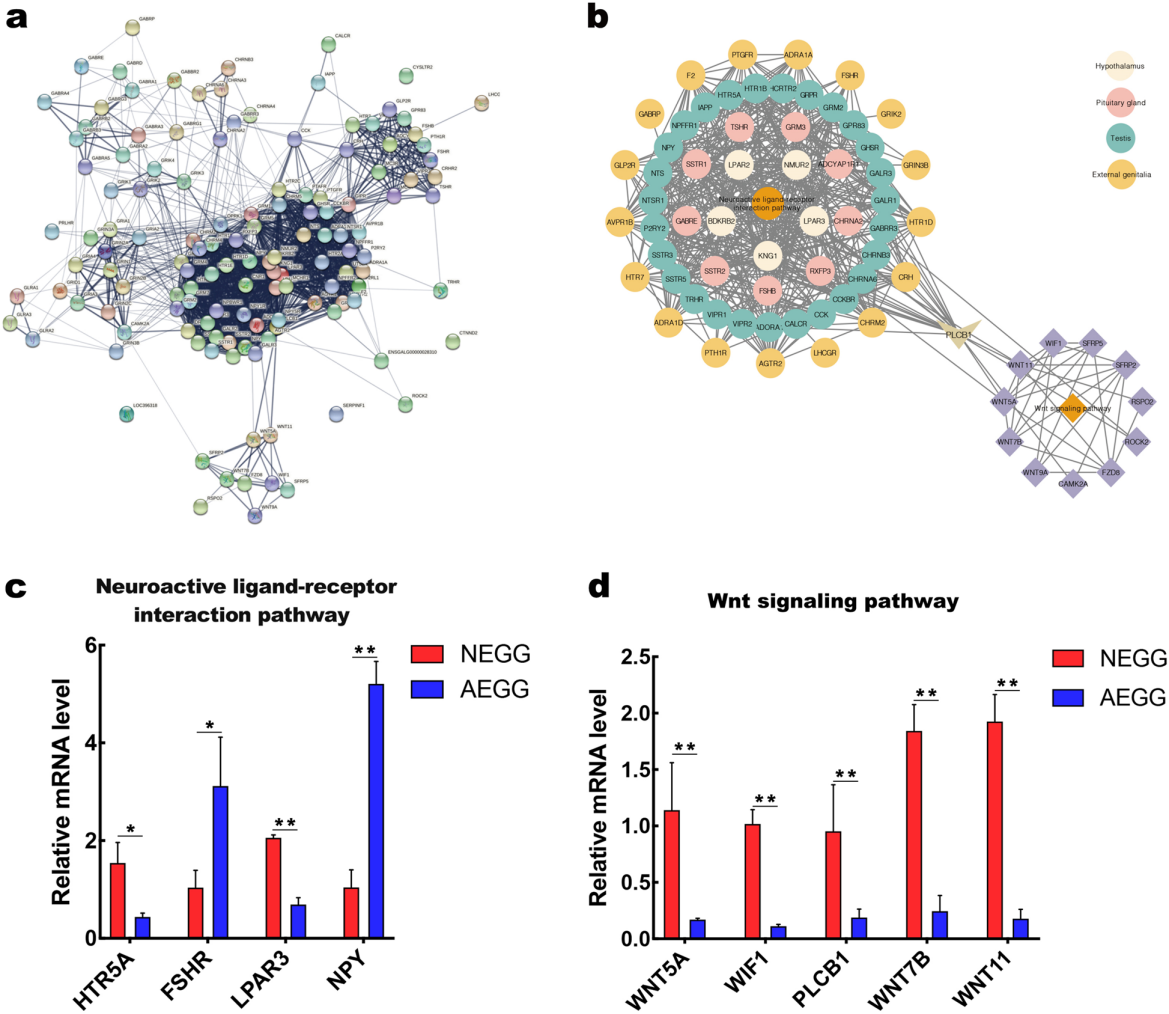
Network analyses of DEGs between NEGG and AEGG. **a** PPI networks of DEGs. The network was constructed by 136 DEGs based on DEGs, WGCNA, KEGG pathway, and GO enrichment, which consisted of 2 significantly important pathways. **b** Regulation network construction involved in external genitalia development of male geese. The circle and diamond represent the neuroactive ligand-receptor interaction pathway and WNT signaling pathway, respectively. **c** qRT-PCR validation of DEGs identified in the neuroactive ligand-receptor interaction pathway. **d** qRT-PCR validation of DEGs identified in the WNT signaling pathway. “*” and “**” represents a significant difference at *P* < 0.05 or *P* < 0.01, respectively

To further identify the hub genes that were associated with the external genitalia development, the DEGs from the hypothalamus, pituitary gland, testis, and external genitalia between NEGG and AEGG were merged to construct the PPI network (Fig. 6a). The PPI network consisted of 133 nodes and 1531 edges. Subsequently, functional analysis revealed that the PPI network were significantly enriched into two pathways (“neuroactive ligand-receptor interaction” and “WNT signaling” pathways). The top highest degree genes included *KNG1*, *LPAR2*, *LPAR3*, *NPY*, *PLCB1*, *AVPR1B*, *GHSR*, *GRM3*, *HTR5A*, *FSHB*, *FSHR*, *WNT11*, *WNT5A*, *WIF1*, and *WNT7B.* Notably, it was postulated that the “neuroactive ligand-receptor interaction” pathway could regulate the “Wnt signaling” pathway through *PLCB1* to control male goose external genitalia development (Fig. 6b). Nine DEGs involved in the external genitalia development were selected for real-time quantitative PCR (qRT-PCR) validation of our RNA-seq results. These included two genes that were up-regulated in the “neuroactive ligand-receptor interaction” pathway (*FSHR,* and *LPAR3*), two genes that were down-regulated in the “neuroactive ligand-receptor interaction” pathway (*HTR5A*, and *NPY*), and five genes that were down-regulated in the “Wnt signaling” pathway (*WNT5A*, *WIF1*, *PLCB1*, *WNT7B*, and *WNT11*). The expression profiles of the nine key genes generated from qRT-PCR corresponded to the RNA-Seq results (Fig. 6c, d), indicating that the RNA-seq results are reliable.

## Discussion

In birds, the HPG axis is a coordinated neuroendocrine system that plays a central role in regulating reproductive functions [18, 19]. A previous study has shown that the external genitalia development of male birds is regulated by different kinds of hormones [20]. In the present study, significant differences in both the length and weight of the external genitalia was observed between the NEGG and AEGG groups. Consistent with this observation, 107, 284, 2192, and 1005 DEGs were identified in the hypothalamus, pituitary gland, testis, and external genitalia between NEGG and AEGG, respectively. Recently, transcriptome studies in poultry HPG axis-related tissues have identified some candidate genes associated with the reproductive system development [17, 21]. Taken together, these identified DEGs can potentially be used to explain the specific functions of HPG axis regulating the development of external genitalia of male goose.

To further reveal the biological implications of these identified DEGs, GO annotation and KEGG enrichment analysis were performed. Most of the DEGs in the hypothalamus were significantly enriched in the GO terms related to collagen trimer, basement membrane, visual perception, and developmental process, suggesting that structural components and developmental process could be essential for the external genitalia development of male geese. The DEGs in the pituitary gland were mainly enriched in integral component of plasma membrane, neuron projection, signal transduction, extracellular matrix, collagen-containing extracellular matrix, extracellular space, calcium ion binding, and heparin binding. Several DEGs including *FSHB*, *HPCA*, *NDOR1*, *KCNJ5*, and *GPD1L* have been reported to be associated with reproductive performance. Expression levels of *FSHβ* were closely related to the serum levels of some reproductive hormones and gonadal development in male [22, 23]. KEGG analysis showed that the DEGs identified in the pituitary gland between NEGG and AEGG were mainly enriched in ECM-receptor interaction, focal adhesion, neuroactive ligand-receptor interaction, MAPK signaling, cytokine-cytokine receptor interaction, p53 signaling, and GnRH signaling pathways. In chickens, the neuroactive ligand-receptor interaction signaling pathway have recently been identified in ovaries, and it could regulate early ovary development and sexual maturity [24].

Most of the DEGs in the testis were enriched in the GO terms related to the microtubule-based movement, microtubule binding, flagellated sperm motility, mitotic cytokinesis, neuropeptide signaling pathway, microtubule motor activity, and integral component of plasma membrane. Several DEGs including *FOXA2*, *SIX2*, *SNTN*, *VSTM2A*, *TDGF1*, *APOB*, and *WNT6* were found to be up-regulated in AEGG geese. Moreover, the DEGs in the testis were mainly enriched into 7 KEGG pathways, including neuroactive ligand-receptor interaction, cell cycle, purine metabolism, oocyte meiosis, calcium signaling pathway, WNT signaling pathway, and progesterone-mediated oocyte maturation, which were important for the external genitalia development. Previous studies have shown that the cell cycle and WNT signaling pathways played important roles in the regulation of mammalian genitalia development [25, 26].

In the external genitalia, most of DEGs were enriched in integral component of membrane, extracellular space, plasma membrane, cell surface, calcium ion binding, integral component of plasma membrane, and cell differentiation. Numerous studies have shown that cell cycle and cell differentiation play an important role in the development of external genitalia [25, 27]. Expression levels of *BMP3*, *HSPA2*, *SKI1*, *WNT9A*, *AGRN*, *WNT5A*, *WNT7B*, *CHRM3*, *MSTN*, *FSHR*, and *LHCGR* genes were involved in the development of genitalia. The DEGs in the external genitalia were mainly enriched into metabolic, neuroactive ligand-receptor interaction, calcium signaling, WNT signaling, adrenergic signaling in cardiomyocytes, ECM-receptor interaction, and purine metabolism pathways. Previous studies have shown that the *BMP* gene could negatively affect proximodistally oriented outgrowth of external genitalia tubercle with regulatory functions on cell proliferation and apoptosis [27]. Moreover, expression of some genes involved in the Wnt signaling pathway (*WNT5A, WNT7A*, and *WNT9A*) are regulated by both estrogen and androgen to direct the proliferation and elongation of the genitalia during differentiation [26].

In addition, results of co-expression network analysis suggested that the “neuroactive ligand-receptor interaction” and “WNT signaling” pathways could play a critical role in affecting male goose external genitalia development. In the present study, the neuroactive ligand-receptor interaction pathway was significantly enriched by both the DEGs identified in the pituitary gland, testis, and external genitalia between NEGG and AEGG groups. Transcriptome studies in poultry [16], goat [28], and yellow croaker fish [29] have also demonstrated the important role of this pathway in the control of reproductive activities. Furthermore, almost all DEGs enriched in this pathway, including *KNG1*, *LPAR2*, *LPAR3*, *NPY*, *AVPR1B*, *GHSR*, *GRM3*, *HTR5A*, *FSHB*, and *FSHR*, were significantly up- or down-regulated in the pituitary gland, testis, and external genitalia between NEGG and AEGG groups. It is well known that *FSHβ*, *FSHR*, and *GHSR* are directly related to hormonal synthesis. *LPAR3* could interact with multiple reproductive hormones, including progesterone [30], and estrogen [31], to affect reproduction performance. Previous reports have shown that altering estrogen levels in males led to abnormal genitalia phenotypes [8, 26]. Furthermore, we found that the WNT signaling pathway could play a significant role during external genitalia development in male geese. In this pathway, *PLCB1*, *WIF1*, *WNT5A*, *WNT11*, and *WNT7B* were downregulated in the AEGG group. Significantly, we found that the neuroactive ligand-receptor interaction pathway could regulate the WNT signaling pathway through *PLCB1*. In recent years, studies in male animals have also demonstrated the important role of the WNT signaling pathway in the control of genitalia development [32–34]. During the external genitalia development, the expression levels of *WNT5A, WNT7A*, and *WNT9A* were tightly regulated by hormones and are critical for its development [26]. As a WNT inhibitory factor, WIF1 was shown to inhibit the WNT/*β*-catenin signaling pathway by binding to WNT molecules to regulate the development of external genitalia [35]. Taken together, our results suggested that the neuroactive ligand-receptor interaction pathway may regulate the WNT signaling pathway through *PLCB1* to control the external genitalia development of male goose.

## Conclusions

In conclusion, the genome-wide transcriptomic profiles in the hypothalamus, pituitary gland, testis, and external genitalia between the NEGG and AEGG groups were compared using RNA-seq. Bioinformatic analysis and validation experiments suggested that these identified DEGs in both the neuroactive ligand-receptor interaction and WNT signaling pathways were crucial for male goose external genitalia development. Furthermore, the neuroactive ligand-receptor interaction pathway could regulate the WNT signaling pathway through *PLCB1* to control the external genitalia development. These results provide novel insights into the mechanisms regulating the external genitalia development of male goose.

## Methods

### Experiment animals and sample collection

The NEGG and AEGG geese were obtained from the Sichuan Agricultural University Waterfowls Breeding Farm (Ya’an, Sichuan, China). All of these geese were provided with free access to feed and water under natural light and temperature condition. At the age of 245 days, the external genitalia length was measured from the top of the external genitalia to the anus using a vernier caliper. When the length of the external genitalia was greater than 4.07 cm, male individuals were classified into the normal external genital group (NEGG); in contrast, those with the external genitalia length less than 2.21 cm were classified into the abnormal external genitalia group (AEGG). In each group, three geese, with similar body weights and physiology conditions were selected for tissue sample collection. All selected geese were euthanized by inhaling carbon dioxide and cervical dislocation, which performed by competent personnel who experienced and correctly applied the technique. Then, the hypothalamus, pituitary gland, testis and external genitalia removed immediately after slaughter. Testis and external genitalia weights were collected quickly. All tissues were washed with phosphate buffered saline (PBS) and frozen in liquid nitrogen, and then stored at -80 °C until RNA extraction.

### RNA isolation and sequencing

For total RNA sequencing, total RNA from hypothalamus, pituitary gland, testis and external genitalia for each individual (a total of 24 samples) were extracted with the using RNeasy Mini Kit (QIAGEN, Beijing, China) following the manufacturer’s instructions. RNA integrity was checked by Agilent Bioanalyzer 2100 (Agilent Technologies, Santa Clara, CA, USA). Samples with average RIN value of 8.96 (from 7.9 to 9.7) were then sent to generate libraries by Novogene (Novogene, Tianjin, China) (Additional file 7: Table S6). All libraries were sequenced by the Novogene Illumina PE 150. The clean reads were obtained after the filtration of low-quality reads using standard quality control by FastaQC software.

### Transcriptome alignment and assembly

Clean reads were mapped against goose reference genome that includes the mitochondrial genome (AnsCyg_PRJ-NA183603_v1.0) using the HISAT2 (version 2.1.0) software [36]. The output SAM (sequencing alignment/mapping) file was converted to a BAM (binary alignment/mapping) file and sorted using SAMtools (version 1.13) [37]. The expression values (fragments per kilobase of transcript per million fragments mapped) of each gene were calculated based on the length of the gene and the read count mapped to this gene by featureCounts (version 2.02) [38].

### Identification of the DEGs and functions analysis

According to the above groups (NEGG and AEGG) based on the length of external genitalia, we performed normalization and differential gene expression analysis using the DESeq2 [39]. False Discovery Rate (FDR) was used as corrected *p*-values (≤ 0.05) due to multiple analysis. The DEGs was filtered based on *p*-values < 0.05 and |log2FC| > 1. KOBAS 3.0 [40] was used to analyze the Gene Ontology (GO) functions and the Kyoto Encyclopedia of Genes and Genomes (KEGG) functions. The functional gene analysis was performed based on *G. gallus* reference. WGCNA package in R was used to construct the co-expression network for all genes in different tissues [41]. The STRING 10 database (http://string-db.org/) was employed to identify the relationship between the DEGs identified in this study. All the network visualization was performed using Cytoscape (version 3.2.1) [42].

### Real-time PCR verification

Nine significantly DEGs were selected for qRT-PCR to validate the RNA-Seq results. Previously, total RNA extracted from the testis and external genitalia were reverse transcribed into cDNA using a RevertAid First Strand cDNA Synthesis Kit (Thermo, MA, USA). Primer 5.0 was used to design the primers (Table 1). A BLAST search against the reference genome was then carried out to confirm primers were specific for the intended target genes. SYBR Green PCR Super Mix (Bio-Rad, Hercules, CA, USA) and a Bio-Rad CFX96 real-time PCR detection system (Bio-Rad, Hercules, CA, USA) were used for qRT-PCR, and each sample was assayed three times. The reaction was performed at 95°C for 10 s, 60°C for 60 s, and 95°C for 15 s, after which it was slowly heated from 60 °C to 99 °C. The *GAPDH* was used as a housekeeping gene. The 2^−ΔΔCt^ method [43] was used for normalization of the qRT-PCR results, after which the normalized data was used for statistical analysis, and *P* < 0.05 was considered significantly different.

**Table 1:** PCR primers used in this study

Gene name	Sequence (5'-3')	Product length (bp)
*HTR5A*-F	CCTGTGCGTGGTGCTTTTTGTG	251
*HTR5A*-R	CGAAGACCCCGATAAGGATGCC	
*FSHR*-F	GGACAACGATGTTCCCAGTGATAG	116
*FSHR*-R	ATGTGCCTTGCTCACCTAAACCT	
*LPAR3*-F	CTGGAGCGTCCTAAACCTTG	368
*LPAR3*-R	CAGACGAGCAGCAAAGCATC	
*NPY*-F	GTGTCGGTGCTGACTTTCGC	160
*NPY*-R	GCCTGGTGATGAGGTTGATGTAG	
*WNT5A*-F	GCTCTTGGTGGTCTTTAGGGATG	470
*WNT5A*-R	CTCCTTGGCGAAGCGGTATC	
*WIF1*-F	CTCCAGACCCCTCAGAACGC	235
*WIF1*-R	TGTCGCAGTTGATGCCGTAG	
*PLCB1*-F	CGGCAAGTGCTTTTGTCAGG	106
*PLCB1*-R	CAGTTGTCATAGTGAAACCGTGTG	
*WNT7B*-F	GCGTTCACCTATGCCATTACTGC	128
*WNT7B*-R	CAGCCCTCCTCCTGGTTGTAGTAG	
*WNT11*-F	GCACAACAGTGAAGTAGGGAGAC	414
*WNT11*-R	CACCGCTCCACCACTTTGTC	
*GAPDH*-F	GTGGTGCAAGAGGCATTGCTGAC	86
*GAPDH*-R	GCTGATGCTCCCATGTTCGTGAT	

### Statistics analysis

Statistical analysis was performed using the SPSS 23.0 software (IBM, USA). The means of the weight and length for external genitalia between NEGG and AEGG were subjected to ANOVA testing, the means were assessed for significance by Tukey’s test, and t-tests was used to analyze the significance between the two groups. Differences were considered statistically significant at *P* < 0.05.

## Supplementary Information

**Additional file 1: Table S1.** The reads account and mapping rate of samples.

**Additional file 2: Figure S1.** Volcanic map of differentially expressed genes. (**a**). NEGG-vs-AEGG hypothalamus; (**b**). NEGG-vs-AEGG pituitary gland; (**c**). NEGG-vs-AEGG testis; (**d**). NEGG-vs-AEGG external genitalia. The abscissa represents the fold change in the expression of the gene in different tissues; The ordinate represents the statistical significance of the difference in the amount of gene expression; The red dot in the figure indicates the up-regulated gene with significant differential expression, and the blue dot indicates the down-regulated gene with significant differential expression.

**Additional file 3: Table S2.** The differentially expressed genes in pituitary glandbetween NEGG and AEGG.

**Additional file 4: Table S3.** Top 30 GO terms enriched by the DEGs in different tissues and related information.

**Additional file 5: Table S4.** Top 30 KEGG pathways enriched by the DEGs in different tissues and related information.

**Additional file 6: Table S5.** The DEGs expression module analysis.

**Additional file 7: Table S6.** Sample quality control.

## Data Availability

All data generated or analyzed during this study are included in this published article and its additional files, or in the following public repositories. The transcriptome sequence data has been submitted to the NCBI Sequence Read Archive repository under the following accession numbers: [PRJNA764457] (https://www.ncbi.nlm.nih.gov/bioproject/764457).

## Abbreviations

AEGG: Abnormal external genitalia group
*Bmp4*: Bone morphogenetic protein 4
*CYP17*: 17α-hydroxylase/17, 20-lyase
DEGs: Differentially expressed genes
FDR: False Discovery Rate
GO: Gene Ontology
*IGF1*: Insulin growth factor 1
KEGG: Kyoto Encyclopedia of Genes and Genomes
NEGG: Normal external genital group
PPI: Protein-protein interaction
*Shh*: Sonic hedgehog
WGCNA: Weighted gene co-expression network analysis
