# Supplementary material for: Comparative transcriptome analysis identifies crucial candidate genes and pathways in the hypothalamic-pituitary-gonadal axis during external genitalia development of male geese
Source: BMC Genomics. 2022 Feb 15;23:136. doi: 10.1186/s12864-022-08374-2 (PMC8848681; doi:10.1186/s12864-022-08374-2)

**Figure legends**

**Additional file 2: Figure S1:** Volcanic map of differentially expressed genes. (**a).** NEGG-vs-AEGG hypothalamus; (**b).** NEGG-vs-AEGG pituitary gland; (**c).** NEGG-vs-AEGG testis; (**d).** NEGG-vs-AEGG external genitalia. The abscissa represents the fold change in the expression of the gene in different tissues; The ordinate represents the statistical significance of the difference in the amount of gene expression; The red dot in the figure indicates the up-regulated gene with significant differential expression, and the blue dot indicates the down-regulated gene with significant differential expression.

**Additional file 2 Figure S1.**


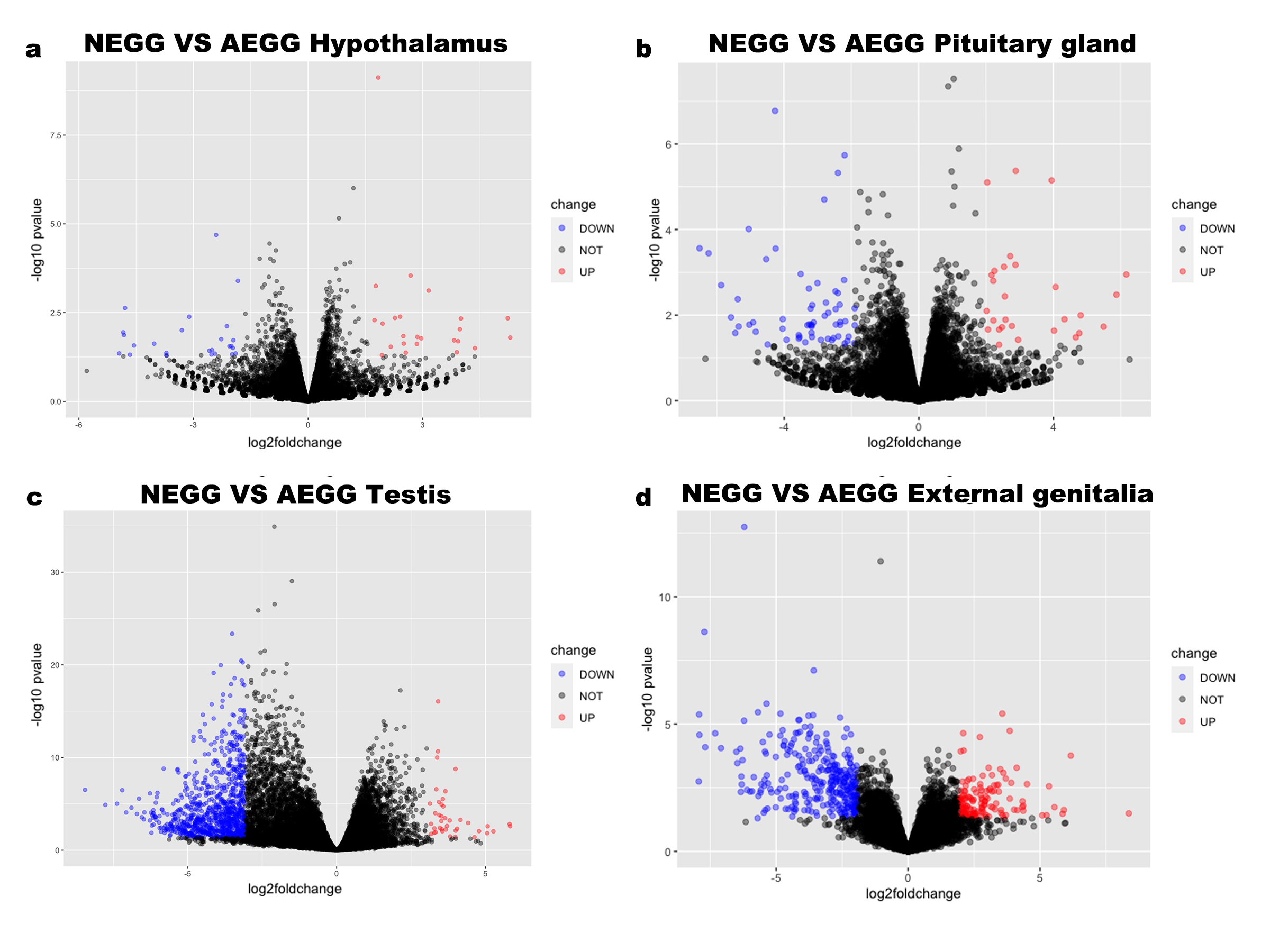

Supplement: Supplementary file 2 — Additional file 2: Figure S1. Volcanic map of differentially expressed genes. (a). NEGG-vs-AEGG hypothalamus; (b). NEGG-vs-AEGG pituitary gland; (c). NEGG-vs-AEGG testis; (d). NEGG-vs-AEGG external genitalia. The abscissa represents the fold change in the expression of the gene in different tissues; The ordinate represents the statistical significance of the difference in the amount of gene expression; The red dot in the figure indicates the up-regulated gene with significant differential expression, and the blue dot indicates the down-regulated gene with significant differential expression. [file 12864_2022_8374_MOESM2_ESM.docx]
